# 3-Benzyl-3-hy­droxy-2-phenyl-3*H*-indole 1-oxide

**DOI:** 10.1107/S1600536810026176

**Published:** 2010-07-10

**Authors:** Corrado Rizzoli, Paola Astolfi, Elda Marku, Lucedio Greci

**Affiliations:** a Dipartimento di Chimica Generale ed Inorganica, Chimica Analitica, Chimica Fisica, Universitá degli Studi di Parma, Viale G. P. Usberti 17/A, I-43100 Parma, Italy; b Dipartimento ISAC, Universitá Politecnica delle Marche, Via Brecce Bianche, I-60131 Ancona, Italy; c Fakulteti i Shkencave të Natyrës, Departamenti i Kimise, Universiteti i Tiranes, Bulevardi "Zogu I", Tirana, Albania

## Abstract

The asymmetric unit of the title compound, C_21_H_17_NO_2_, contains two crystallographically independent mol­ecules of similar geometry. The indole ring systems form dihedral angles of 8.30 (5) and 9.58 (5)° with the attached phenyl rings, and 56.96 (5) and 57.68 (5)° with the aromatic rings of the respective benzyl groups. The mol­ecular conformations are stabilized by intra­molecular C—H⋯O hydrogen bonds. In the crystal structure, centrosymmetrically related pairs of mol­ecules are linked into dimers through pairs of inter­molecular O—H⋯O hydrogen bonds, generating 12-membered rings with *R*_2_^2^(12) motifs. The dimers are further linked into a three-dimensional network by C—H⋯O inter­actions.

## Related literature

For the use of nitro­nes in the spin-trapping technique and in organic synthesis, see: Janzen (1971 ▶); Zubarev (1979 ▶); Balasubramanian (1985 ▶); Pisaneschi *et al.* (2002 ▶); Jones *et al.* (2000 ▶); Bernotas *et al.* (1999 ▶); Ali & Wazeer (1988 ▶); Merino (2005 ▶); Chiacchio *et al.* (2006 ▶); Revuelta *et al.* (2008 ▶); Astolfi *et al.* (2003 ▶); Greci *et al.* (2001 ▶); Tommasi *et al.* (1999 ▶); Bruni *et al.* (1998 ▶). For a related structure, see: Yamada *et al.* (2003 ▶). For graph-set notation, see: Bernstein *et al.* (1995 ▶). For the preparation of 2-phenyl­isatogen, see: Bond & Hooper (1974 ▶).
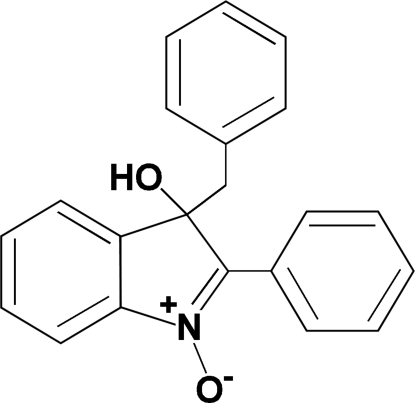


## Experimental

### 

#### Crystal data

C_21_H_17_NO_2_
*M* *_r_* = 315.36
Triclinic, 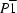

*a* = 11.635 (2) Å
*b* = 11.971 (2) Å
*c* = 12.063 (3) Å
α = 84.773 (5)°
β = 88.882 (6)°
γ = 88.635 (6)°
*V* = 1672.5 (6) Å^3^
*Z* = 4
Cu *K*α radiation
μ = 0.64 mm^−1^
*T* = 294 K
0.26 × 0.24 × 0.18 mm

#### Data collection

Siemens AED diffractometer
6045 measured reflections
6045 independent reflections
5126 reflections with *I* > 2σ(*I*)
3 standard reflections every 100 reflections  intensity decay: 0.02%

#### Refinement

*R*[*F* ^2^ > 2σ(*F* ^2^)] = 0.053
*wR*(*F* ^2^) = 0.162
*S* = 1.05
6045 reflections
442 parameters
H atoms treated by a mixture of independent and constrained refinement
Δρ_max_ = 0.22 e Å^−3^
Δρ_min_ = −0.24 e Å^−3^

### 

Data collection: *AED* (Belletti *et al.*, 1993 ▶); cell refinement: *AED*; data reduction: *AED*; program(s) used to solve structure: *SIR97* (Altomare *et al.*, 1999 ▶); program(s) used to refine structure: *SHELXL97* (Sheldrick, 2008 ▶); molecular graphics: *ORTEP-3 for Windows* (Farrugia, 1997 ▶) and *SCHAKAL97* (Keller, 1997 ▶); software used to prepare material for publication: *SHELXL97* and *PARST95* (Nardelli, 1995 ▶).

## Supplementary Material

Crystal structure: contains datablocks global, I. DOI: 10.1107/S1600536810026176/ez2222sup1.cif

Structure factors: contains datablocks I. DOI: 10.1107/S1600536810026176/ez2222Isup2.hkl

Additional supplementary materials:  crystallographic information; 3D view; checkCIF report

## Figures and Tables

**Table 1:** Hydrogen-bond geometry (Å, °)

*D*—H⋯*A*	*D*—H	H⋯*A*	*D*⋯*A*	*D*—H⋯*A*
C10—H10⋯O1	0.93	2.19	2.817 (3)	124
C14—H14⋯O2	0.93	2.34	2.996 (2)	127
C31—H31⋯O4	0.93	2.47	3.107 (2)	126
C35—H35⋯O3	0.93	2.37	2.989 (3)	124
C11—H11⋯O4	0.93	2.48	3.404 (3)	175
O2—H2*O*⋯O1^i^	0.90 (2)	1.88 (2)	2.769 (2)	174 (2)
O4—H4*O*⋯O3^ii^	0.98 (2)	1.82 (2)	2.793 (2)	178 (2)
C24—H24⋯O1^ii^	0.93	2.48	3.310 (3)	148
C3—H3⋯O3^iii^	0.93	2.46	3.327 (3)	154
C34—H34⋯O2^iv^	0.93	2.49	3.415 (3)	176

Symmetry codes: (i) 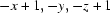; (ii) 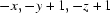; (iii) 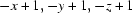; (iv) 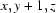.

**Fig. 1.**
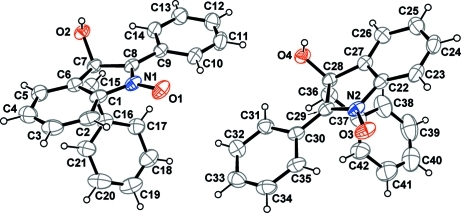
The asymmetric unit of the title compound. Displacement ellipsoids are drawn at the 30% probability level.

**Fig. 2.**
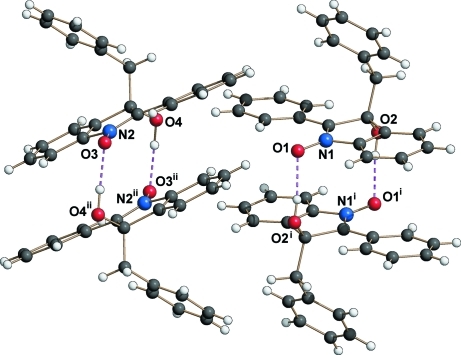
View of the centrosymmetric dimers of the title formed through intermolecular O—H···O hydrogen bonds (dashed lines). Symmetry codes: (i) 1 - x, -y, 1 - z; (ii) -x, 1 - y, 1 - z.

**Fig. 3.**
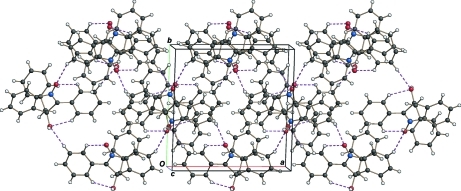
Crystal packing of the title compound viewed approximately along the c axis. Intra- and intermolecular hydrogen bonds are shown as dashed lines.

## supplementary crystallographic information

### Comment

Many types of cyclic and acyclic nitrones, such as *N*-*tert*-butyl-α-phenylnitrone and 5,5-dimethyl-pyrroline-*N*-oxide, have been used frequently in the spin trapping technique since its inception (Janzen, 1971; Zubarev, 1979). Nitrones are also used in the syntheses of isoxazolidines through 1,3-dipolar cycloaddition with a series of dipolarophiles (Balasubramanian, 1985). Although the most used nitrones in cyclization reactions are acyclic, several papers have appeared in the last two decades describing cycloaddition reactions with cyclic nitrones (Pisaneschi *et al.*, 2002; Jones *et al.*, 2000; Bernotas *et al.*, 1999; Ali & Wazeer, 1988). Significant advances have been described in the use of nitrones derived from sugars and aminoacids for the synthesis of interesting biological compounds including aminoacids, amino alcohols and nucleoside analogs (Merino, 2005). On this basis, enantioselective syntheses of homo-carboxylic-2'-oxo-3'-azo-nucleosides were achieved by cycloaddition reactions of *N*-glycosyl nitrones with allylic nucleobases (Chiacchio *et al.*, 2006). Moreover, a series of 3-spirocyclopropane dihydro- and tetrahydropyrid-4-ones were synthesized by nitrone cycloaddition to 1,1'-bicyclopropylidene (Revuelta *et al.*, 2008). The title compound was synthesized in order to continue our studies on 1,3-dipolar cycloaddition with different dipolarophiles, with particular focus on the catalytic activity of metal cations such as cobalt(II), calcium(II), zinc(II) and nickel(II) (Astolfi *et al.*, 2003; Greci *et al.*, 2001; Tommasi *et al.*, 1999; Bruni *et al.*, 1998).

The asymmetric unit of the title compound (Fig. 1) contains two crystallographically independent molecules with similar geometry. The indole ring systems including the N1 and N2 atoms form dihedral angles of 8.30 (5) and 9.58 (5)°, respectively, with the attached phenyl rings, and 56.96 (5) and 57.68 (5)°, respectively, with the aromatic ring of the benzyl groups. The N–O (mean value 1.304 (2) Å) and C–O (mean value 1.420 (2) Å) bond lengths are comparable with those found in 3-hydroxy-2,3-dimethyl-3*H*-indole *N*-oxide [1.3093 (17) and 1.418 (2) Å respectively; Yamada *et al.*, 2003]. The molecular conformations are stabilized by intramolecular C—H···O hydrogen bonds (Table 1). In the crystal packing, centrosymmetrically related molecules are linked into dimers (Fig. 2) through intermolecular O—H···O hydrogen bonds resulting in twelve-membered rings with *R*_2_^2^(12) motifs (Bernstein *et al.*, 1995). Within the dimers, the centroid-to-centroid separations between the opposite C1–C6/C9^i^–C14^i^ and C22–C27/C30^ii^–C35^ii^ aromatic rings are 3.893 (2) and 3.920 (2) Å, respectively (symmetry codes: (i) 1 - *x*. -*y*, 1 - *z*; (ii) -*x*, 1 - *y*, 1 - *z*). The dimers are further connected by C—H···O hydrogen bonds into a three-dimensional network (Fig. 3).

### Experimental

A solution of benzylmagnesium bromide (20 mmoles in 30 ml of dried THF, obtained from 0.46 g of magnesium and 2.54 g of benzyl chloride in a current of argon) was added to a solution of 2-phenylisatogen (10 mmoles, 2.23 g in 50 ml of dried THF; Bond & Hooper, 1974), at room temperature and under magnetic stirring. After the addition, the reaction mixture was kept at room temperature for 2 h, then it was poured into 10% aqueous NH_4_Cl (100 ml) solution. The mixture was extracted with chloroform (2 × 50 ml) and the separated organic layer was dried on Na_2_SO_4_ and evaporated to dryness. The residue was treated with diethyl ether to give a white solid corresponding to the expected nitrone, which was separated by filtration under vacuum and washed with diethyl ether (obtained 2.04 g, yield 65%, m.p. 200–201 °C. FT—IR, ν, cm^-1^, 3143 (OH). 1601 (O<-N=C<), 1519. ^1^H NMR, δ, CDCl_3_: 3.36 (2*H*, pseudo-q, –CH_2_Ph, distereotopic H atoms), 6.41 (2*H*, d, arom.), 6.81–7.07 (5*H*,m, arom.). 7.13–7.55 (3*H*, m, arom.), 7.3–7.4 (2H. m, arom.), 8.6 (2*H*, pseudo-q, arom). Mass. calcd. for C_21_H_17_NO_2_, 315.39; found: *m*/*z* (%): 315 (*M*+, 5.7), 224 (34.4), 208 (58.6), 179 (100). The melting point was measured on a Mitamura Riken Kogyo mp D electrochemical apparartus and was not corrected. FT—IR spectrum was recorded in KBr with a Perkin-Elmer MGX1 spectrophotometer equipped with Spectra Tech. ^1^H NMR spectrum was recorded on a Gemini Varian 200 MHz. Mass spectrum was recorded on a Carlo Erba QMD 1000 mass spectrometer in positive electron impact (EI) mode. Single crystals of the title compound suitable for X-ray analysis were obtained by slow evaporation of an ethanol solution at room temperature.

### Refinement

The hydroxy H atoms were located in a difference Fourier map and refined freely. All other H atoms were placed at calculated positions and refined using a riding model approximation, with C—H = 0.93–0.97 Å, and with *U*_iso_(H) = 1.2 *U*_eq_(C).

### Crystal data

**Table:**

C_21_H_17_NO_2_	*Z* = 4
*M**_r_* = 315.36	*F*(000) = 664
Triclinic, *P*1	*D*_x_ = 1.252 Mg m^−^^3^
Hall symbol: -P 1	Cu *K*α radiation, λ = 1.54178 Å
*a* = 11.635 (2) Å	Cell parameters from 48 reflections
*b* = 11.971 (2) Å	θ = 16.4–48.4°
*c* = 12.063 (3) Å	µ = 0.64 mm^−^^1^
α = 84.773 (5)°	*T* = 294 K
β = 88.882 (6)°	Block, pale yellow
γ = 88.635 (6)°	0.26 × 0.24 × 0.18 mm
*V* = 1672.5 (6) Å^3^	

### Data collection

**Table:**

Siemens AED diffractometer	*R*_int_ = 0.0000
Radiation source: fine-focus sealed tube	θ_max_ = 68.0°, θ_min_ = 3.7°
graphite	*h* = −9→13
θ/2θ scans	*k* = −14→10
6045 measured reflections	*l* = −14→14
6045 independent reflections	3 standard reflections every 100 reflections
5126 reflections with *I* > 2σ(*I*)	intensity decay: 0.02%

### Refinement

**Table:**

Refinement on *F*^2^	Secondary atom site location: difference Fourier map
Least-squares matrix: full	Hydrogen site location: inferred from neighbouring sites
*R*[*F*^2^ > 2σ(*F*^2^)] = 0.053	H atoms treated by a mixture of independent and constrained refinement
*wR*(*F*^2^) = 0.162	*w* = 1/[σ^2^(*F*_o_^2^) + (0.0996*P*)^2^ + 0.2753*P*] where *P* = (*F*_o_^2^ + 2*F*_c_^2^)/3
*S* = 1.05	(Δ/σ)_max_ < 0.001
6045 reflections	Δρ_max_ = 0.22 e Å^−^^3^
442 parameters	Δρ_min_ = −0.24 e Å^−^^3^
0 restraints	Extinction correction: *SHELXL97* (Sheldrick, 2008)
Primary atom site location: structure-invariant direct methods	Extinction coefficient: 0.0034 (5)

### Special details

**Table:**

Refinement. Refinement of *F*^2^ against ALL reflections. The weighted *R*-factor *wR* and goodness of fit *S* are based on *F*^2^, conventional *R*-factors *R* are based on *F*, with *F* set to zero for negative *F*^2^. The threshold expression of *F*^2^ > σ(*F*^2^) is used only for calculating *R*-factors(gt) *etc*. and is not relevant to the choice of reflections for refinement. *R*-factors based on *F*^2^ are statistically about twice as large as those based on *F*, and *R*- factors based on ALL data will be even larger.

### Fractional atomic coordinates and isotropic or equivalent isotropic displacement parameters (Å^2^)

**Table:**

	*x*	*y*	*z*	*U*_iso_*/*U*_eq_	
O1	0.46993 (13)	0.19070 (10)	0.43394 (10)	0.0705 (4)	
O2	0.54286 (11)	−0.16774 (9)	0.33565 (11)	0.0571 (3)	
H2O	0.534 (2)	−0.172 (2)	0.410 (2)	0.088 (7)*	
O3	0.03259 (14)	0.68855 (10)	0.44282 (11)	0.0754 (4)	
O4	−0.00423 (11)	0.33865 (9)	0.32592 (12)	0.0628 (3)	
H4O	−0.013 (2)	0.328 (2)	0.407 (2)	0.097 (8)*	
N1	0.52177 (13)	0.10469 (10)	0.39161 (11)	0.0549 (4)	
N2	−0.00636 (14)	0.60551 (10)	0.39494 (11)	0.0565 (4)	
C1	0.64142 (16)	0.08049 (13)	0.40690 (13)	0.0555 (4)	
C2	0.7148 (2)	0.14275 (16)	0.45982 (15)	0.0696 (5)	
H2	0.6920	0.2085	0.4904	0.084*	
C3	0.8267 (2)	0.10075 (19)	0.46471 (17)	0.0784 (6)	
H3	0.8815	0.1401	0.4995	0.094*	
C4	0.86049 (19)	0.0018 (2)	0.41950 (17)	0.0772 (6)	
H4	0.9363	−0.0239	0.4261	0.093*	
C5	0.78426 (17)	−0.05788 (16)	0.36581 (16)	0.0654 (5)	
H5	0.8061	−0.1236	0.3348	0.079*	
C6	0.67341 (15)	−0.01605 (13)	0.35970 (13)	0.0541 (4)	
C7	0.57370 (14)	−0.05489 (12)	0.30169 (13)	0.0511 (4)	
C8	0.47831 (15)	0.02996 (12)	0.33305 (12)	0.0499 (4)	
C9	0.36031 (15)	0.03120 (13)	0.30073 (13)	0.0537 (4)	
C10	0.28318 (18)	0.12083 (16)	0.31429 (16)	0.0669 (5)	
H10	0.3074	0.1834	0.3471	0.080*	
C11	0.1726 (2)	0.1165 (2)	0.2795 (2)	0.0818 (6)	
H11	0.1214	0.1759	0.2885	0.098*	
C12	0.1379 (2)	0.0260 (2)	0.23201 (19)	0.0826 (6)	
H12	0.0623	0.0235	0.2087	0.099*	
C13	0.21224 (19)	−0.06358 (19)	0.21721 (19)	0.0772 (6)	
H13	0.1866	−0.1253	0.1839	0.093*	
C14	0.32193 (17)	−0.06139 (15)	0.25114 (16)	0.0652 (5)	
H14	0.3719	−0.1217	0.2414	0.078*	
C15	0.60383 (16)	−0.04876 (14)	0.17393 (14)	0.0588 (4)	
H151	0.6698	−0.0979	0.1630	0.071*	
H152	0.5396	−0.0773	0.1357	0.071*	
C16	0.63050 (16)	0.06764 (15)	0.12009 (13)	0.0592 (4)	
C17	0.5463 (2)	0.13279 (18)	0.06942 (16)	0.0734 (5)	
H17	0.4716	0.1067	0.0693	0.088*	
C18	0.5709 (3)	0.2378 (2)	0.0179 (2)	0.0979 (8)	
H18	0.5122	0.2798	−0.0183	0.117*	
C19	0.6775 (3)	0.2818 (2)	0.0181 (2)	0.1036 (9)	
H19	0.6915	0.3532	−0.0160	0.124*	
C20	0.7618 (3)	0.2195 (2)	0.0687 (2)	0.1025 (9)	
H20	0.8356	0.2475	0.0706	0.123*	
C21	0.7385 (2)	0.1121 (2)	0.11877 (17)	0.0803 (6)	
H21	0.7982	0.0693	0.1523	0.096*	
C22	−0.12773 (16)	0.58215 (13)	0.40787 (13)	0.0560 (4)	
C23	−0.2149 (2)	0.63966 (15)	0.46520 (15)	0.0696 (5)	
H23	−0.1988	0.7036	0.5000	0.084*	
C24	−0.3243 (2)	0.59772 (18)	0.46771 (17)	0.0768 (6)	
H24	−0.3838	0.6331	0.5045	0.092*	
C25	−0.34455 (18)	0.5030 (2)	0.41531 (17)	0.0753 (6)	
H25	−0.4183	0.4744	0.4180	0.090*	
C26	−0.25587 (17)	0.44760 (17)	0.35706 (16)	0.0674 (5)	
H26	−0.2717	0.3839	0.3219	0.081*	
C27	−0.14607 (15)	0.48919 (14)	0.35319 (13)	0.0555 (4)	
C28	−0.03287 (15)	0.45255 (13)	0.29552 (14)	0.0534 (4)	
C29	0.05241 (15)	0.53448 (12)	0.33312 (13)	0.0520 (4)	
C30	0.17631 (16)	0.53540 (13)	0.30252 (13)	0.0552 (4)	
C31	0.22917 (17)	0.44455 (16)	0.25119 (16)	0.0667 (5)	
H31	0.1845	0.3840	0.2378	0.080*	
C32	0.34565 (19)	0.4425 (2)	0.22003 (19)	0.0775 (6)	
H32	0.3769	0.3814	0.1865	0.093*	
C33	0.41384 (19)	0.5308 (2)	0.23898 (19)	0.0807 (6)	
H33	0.4914	0.5306	0.2188	0.097*	
C34	0.3640 (2)	0.62003 (19)	0.28893 (19)	0.0802 (6)	
H34	0.4097	0.6801	0.3017	0.096*	
C35	0.24684 (18)	0.62393 (15)	0.32136 (16)	0.0667 (5)	
H35	0.2169	0.6853	0.3552	0.080*	
C36	−0.03763 (17)	0.46461 (16)	0.16720 (15)	0.0658 (5)	
H361	0.0389	0.4511	0.1372	0.079*	
H362	−0.0872	0.4075	0.1436	0.079*	
C37	−0.08058 (18)	0.5768 (2)	0.11951 (14)	0.0698 (5)	
C38	−0.1895 (2)	0.5879 (3)	0.0830 (2)	0.1048 (9)	
H38	−0.2381	0.5270	0.0890	0.126*	
C39	−0.2281 (3)	0.6934 (4)	0.0360 (3)	0.1300 (13)	
H39	−0.3022	0.7011	0.0083	0.156*	
C40	−0.1597 (4)	0.7850 (3)	0.0297 (2)	0.1215 (12)	
H40	−0.1878	0.8545	0.0001	0.146*	
C41	−0.0534 (3)	0.7740 (3)	0.0660 (2)	0.1153 (10)	
H41	−0.0058	0.8356	0.0616	0.138*	
C42	−0.0136 (2)	0.6711 (2)	0.11025 (19)	0.0886 (7)	
H42	0.0616	0.6644	0.1351	0.106*	

### Atomic displacement parameters (Å^2^)

**Table:**

	*U*^11^	*U*^22^	*U*^33^	*U*^12^	*U*^13^	*U*^23^
O1	0.1094 (11)	0.0420 (6)	0.0622 (7)	0.0093 (6)	−0.0094 (7)	−0.0169 (5)
O2	0.0737 (8)	0.0372 (5)	0.0614 (7)	−0.0032 (5)	−0.0124 (6)	−0.0074 (5)
O3	0.1173 (11)	0.0449 (6)	0.0674 (8)	−0.0154 (7)	−0.0035 (7)	−0.0198 (6)
O4	0.0762 (8)	0.0421 (6)	0.0721 (8)	−0.0051 (5)	0.0043 (6)	−0.0153 (5)
N1	0.0815 (10)	0.0375 (6)	0.0462 (7)	−0.0018 (6)	−0.0049 (6)	−0.0057 (5)
N2	0.0861 (10)	0.0369 (6)	0.0472 (7)	−0.0038 (6)	−0.0050 (7)	−0.0061 (5)
C1	0.0762 (11)	0.0465 (8)	0.0440 (8)	−0.0124 (8)	−0.0081 (7)	−0.0009 (6)
C2	0.0989 (15)	0.0541 (10)	0.0573 (10)	−0.0246 (10)	−0.0130 (10)	−0.0049 (8)
C3	0.0884 (15)	0.0805 (14)	0.0672 (12)	−0.0354 (12)	−0.0210 (10)	0.0015 (10)
C4	0.0696 (12)	0.0928 (15)	0.0682 (12)	−0.0163 (11)	−0.0189 (9)	0.0065 (11)
C5	0.0699 (11)	0.0632 (10)	0.0631 (10)	−0.0044 (9)	−0.0128 (9)	−0.0018 (8)
C6	0.0659 (10)	0.0487 (8)	0.0481 (8)	−0.0080 (7)	−0.0076 (7)	−0.0033 (6)
C7	0.0633 (10)	0.0385 (7)	0.0526 (9)	−0.0030 (7)	−0.0087 (7)	−0.0076 (6)
C8	0.0681 (10)	0.0379 (7)	0.0439 (8)	−0.0024 (7)	−0.0035 (7)	−0.0044 (6)
C9	0.0639 (10)	0.0481 (8)	0.0477 (8)	0.0011 (7)	−0.0040 (7)	0.0022 (6)
C10	0.0798 (13)	0.0537 (10)	0.0663 (11)	0.0101 (9)	−0.0030 (9)	−0.0034 (8)
C11	0.0777 (14)	0.0766 (14)	0.0881 (15)	0.0244 (11)	−0.0061 (11)	0.0034 (11)
C12	0.0699 (13)	0.0929 (16)	0.0826 (14)	0.0056 (11)	−0.0164 (11)	0.0074 (12)
C13	0.0748 (13)	0.0748 (13)	0.0827 (14)	−0.0044 (10)	−0.0230 (11)	−0.0054 (10)
C14	0.0683 (11)	0.0548 (10)	0.0729 (11)	0.0040 (8)	−0.0137 (9)	−0.0072 (8)
C15	0.0707 (11)	0.0566 (9)	0.0514 (9)	−0.0022 (8)	−0.0059 (8)	−0.0162 (7)
C16	0.0731 (11)	0.0647 (10)	0.0410 (8)	−0.0062 (8)	−0.0036 (7)	−0.0099 (7)
C17	0.0818 (13)	0.0790 (13)	0.0582 (10)	−0.0040 (10)	−0.0081 (9)	0.0018 (9)
C18	0.120 (2)	0.0863 (16)	0.0831 (16)	0.0026 (15)	−0.0070 (14)	0.0172 (13)
C19	0.150 (3)	0.0783 (15)	0.0802 (16)	−0.0247 (17)	−0.0084 (16)	0.0105 (12)
C20	0.117 (2)	0.112 (2)	0.0792 (15)	−0.0545 (17)	−0.0068 (14)	0.0015 (14)
C21	0.0802 (14)	0.0930 (15)	0.0670 (12)	−0.0187 (12)	−0.0094 (10)	0.0032 (11)
C22	0.0791 (11)	0.0433 (8)	0.0445 (8)	0.0054 (7)	−0.0003 (7)	0.0000 (6)
C23	0.1010 (16)	0.0503 (9)	0.0556 (10)	0.0192 (10)	0.0048 (10)	−0.0009 (7)
C24	0.0853 (14)	0.0756 (13)	0.0647 (11)	0.0298 (11)	0.0087 (10)	0.0088 (10)
C25	0.0662 (12)	0.0928 (15)	0.0640 (11)	0.0098 (10)	−0.0006 (9)	0.0066 (10)
C26	0.0694 (11)	0.0719 (12)	0.0611 (10)	−0.0019 (9)	−0.0027 (9)	−0.0058 (9)
C27	0.0680 (10)	0.0522 (9)	0.0464 (8)	0.0010 (7)	−0.0018 (7)	−0.0057 (7)
C28	0.0637 (10)	0.0451 (8)	0.0529 (9)	−0.0041 (7)	−0.0012 (7)	−0.0121 (7)
C29	0.0722 (10)	0.0395 (7)	0.0446 (8)	−0.0036 (7)	−0.0050 (7)	−0.0048 (6)
C30	0.0697 (11)	0.0474 (8)	0.0483 (8)	−0.0096 (7)	−0.0100 (7)	0.0013 (7)
C31	0.0690 (11)	0.0613 (10)	0.0711 (11)	−0.0095 (9)	−0.0002 (9)	−0.0101 (9)
C32	0.0707 (12)	0.0822 (14)	0.0799 (13)	−0.0007 (10)	−0.0004 (10)	−0.0089 (11)
C33	0.0653 (12)	0.0951 (16)	0.0788 (13)	−0.0150 (11)	−0.0116 (10)	0.0137 (12)
C34	0.0828 (14)	0.0735 (13)	0.0830 (14)	−0.0281 (11)	−0.0235 (11)	0.0119 (11)
C35	0.0826 (13)	0.0528 (9)	0.0647 (11)	−0.0149 (9)	−0.0196 (9)	0.0027 (8)
C36	0.0715 (11)	0.0758 (12)	0.0534 (10)	−0.0076 (9)	−0.0023 (8)	−0.0229 (9)
C37	0.0718 (12)	0.0986 (15)	0.0402 (8)	0.0051 (10)	−0.0028 (8)	−0.0143 (9)
C38	0.0843 (16)	0.148 (3)	0.0839 (16)	0.0134 (16)	−0.0179 (13)	−0.0224 (16)
C39	0.101 (2)	0.193 (4)	0.095 (2)	0.056 (3)	−0.0255 (17)	−0.017 (2)
C40	0.139 (3)	0.143 (3)	0.0747 (16)	0.051 (2)	0.0073 (18)	0.0141 (18)
C41	0.138 (3)	0.109 (2)	0.0904 (18)	0.0069 (19)	−0.0045 (17)	0.0307 (16)
C42	0.0984 (17)	0.0911 (16)	0.0722 (13)	−0.0070 (13)	−0.0114 (12)	0.0182 (11)

### Geometric parameters (Å, °)

**Table:**

O1—N1	1.3186 (18)	C19—C20	1.341 (4)
O2—C7	1.4276 (18)	C19—H19	0.9300
O2—H2O	0.90 (3)	C20—C21	1.400 (3)
O3—N2	1.2898 (17)	C20—H20	0.9300
O4—C28	1.413 (2)	C21—H21	0.9300
O4—H4O	0.98 (3)	C22—C27	1.367 (2)
N1—C8	1.306 (2)	C22—C23	1.417 (3)
N1—C1	1.427 (2)	C23—C24	1.378 (3)
N2—C29	1.347 (2)	C23—H23	0.9300
N2—C22	1.449 (2)	C24—C25	1.375 (3)
C1—C2	1.353 (2)	C24—H24	0.9300
C1—C6	1.375 (2)	C25—C26	1.422 (3)
C2—C3	1.385 (3)	C25—H25	0.9300
C2—H2	0.9300	C26—C27	1.381 (3)
C3—C4	1.394 (3)	C26—H26	0.9300
C3—H3	0.9300	C27—C28	1.550 (2)
C4—C5	1.361 (3)	C28—C29	1.516 (2)
C4—H4	0.9300	C28—C36	1.543 (2)
C5—C6	1.373 (3)	C29—C30	1.481 (3)
C5—H5	0.9300	C30—C35	1.393 (2)
C6—C7	1.473 (2)	C30—C31	1.421 (3)
C7—C8	1.551 (2)	C31—C32	1.400 (3)
C7—C15	1.569 (2)	C31—H31	0.9300
C8—C9	1.434 (2)	C32—C33	1.375 (3)
C9—C14	1.393 (2)	C32—H32	0.9300
C9—C10	1.401 (2)	C33—C34	1.383 (3)
C10—C11	1.365 (3)	C33—H33	0.9300
C10—H10	0.9300	C34—C35	1.412 (3)
C11—C12	1.344 (3)	C34—H34	0.9300
C11—H11	0.9300	C35—H35	0.9300
C12—C13	1.384 (3)	C36—C37	1.491 (3)
C12—H12	0.9300	C36—H361	0.9700
C13—C14	1.349 (3)	C36—H362	0.9700
C13—H13	0.9300	C37—C38	1.350 (3)
C14—H14	0.9300	C37—C42	1.382 (3)
C15—C16	1.519 (2)	C38—C39	1.403 (5)
C15—H151	0.9700	C38—H38	0.9300
C15—H152	0.9700	C39—C40	1.366 (5)
C16—C17	1.357 (3)	C39—H39	0.9300
C16—C21	1.375 (3)	C40—C41	1.319 (5)
C17—C18	1.384 (3)	C40—H40	0.9300
C17—H17	0.9300	C41—C42	1.371 (4)
C18—C19	1.359 (4)	C41—H41	0.9300
C18—H18	0.9300	C42—H42	0.9300
			
C7—O2—H2O	106.0 (15)	C21—C20—H20	120.1
C28—O4—H4O	106.1 (14)	C16—C21—C20	122.4 (2)
C8—N1—O1	128.88 (16)	C16—C21—H21	118.8
C8—N1—C1	109.39 (14)	C20—C21—H21	118.8
O1—N1—C1	121.73 (14)	C27—C22—C23	124.02 (19)
O3—N2—C29	128.03 (17)	C27—C22—N2	106.63 (15)
O3—N2—C22	118.08 (14)	C23—C22—N2	129.34 (16)
C29—N2—C22	113.89 (13)	C24—C23—C22	117.77 (19)
C2—C1—C6	123.80 (19)	C24—C23—H23	121.1
C2—C1—N1	125.66 (18)	C22—C23—H23	121.1
C6—C1—N1	110.54 (14)	C25—C24—C23	119.37 (19)
C1—C2—C3	114.6 (2)	C25—C24—H24	120.3
C1—C2—H2	122.7	C23—C24—H24	120.3
C3—C2—H2	122.7	C24—C25—C26	121.8 (2)
C2—C3—C4	122.57 (18)	C24—C25—H25	119.1
C2—C3—H3	118.7	C26—C25—H25	119.1
C4—C3—H3	118.7	C27—C26—C25	119.44 (19)
C5—C4—C3	121.1 (2)	C27—C26—H26	120.3
C5—C4—H4	119.5	C25—C26—H26	120.3
C3—C4—H4	119.5	C22—C27—C26	117.58 (17)
C4—C5—C6	116.65 (19)	C22—C27—C28	109.74 (15)
C4—C5—H5	121.7	C26—C27—C28	132.67 (16)
C6—C5—H5	121.7	O4—C28—C29	114.21 (14)
C5—C6—C1	121.32 (16)	O4—C28—C36	105.78 (13)
C5—C6—C7	130.23 (16)	C29—C28—C36	109.38 (14)
C1—C6—C7	108.35 (15)	O4—C28—C27	111.76 (14)
O2—C7—C6	114.25 (13)	C29—C28—C27	102.27 (13)
O2—C7—C8	111.51 (13)	C36—C28—C27	113.64 (15)
C6—C7—C8	101.63 (12)	N2—C29—C30	127.86 (15)
O2—C7—C15	107.19 (12)	N2—C29—C28	107.32 (15)
C6—C7—C15	108.21 (14)	C30—C29—C28	124.79 (14)
C8—C7—C15	114.13 (13)	C35—C30—C31	116.51 (18)
N1—C8—C9	123.70 (15)	C35—C30—C29	122.62 (17)
N1—C8—C7	109.85 (14)	C31—C30—C29	120.87 (15)
C9—C8—C7	126.41 (13)	C32—C31—C30	122.72 (18)
C14—C9—C10	118.79 (17)	C32—C31—H31	118.6
C14—C9—C8	117.56 (15)	C30—C31—H31	118.6
C10—C9—C8	123.64 (16)	C33—C32—C31	119.9 (2)
C11—C10—C9	120.11 (19)	C33—C32—H32	120.0
C11—C10—H10	119.9	C31—C32—H32	120.0
C9—C10—H10	119.9	C32—C33—C34	118.2 (2)
C12—C11—C10	119.8 (2)	C32—C33—H33	120.9
C12—C11—H11	120.1	C34—C33—H33	120.9
C10—C11—H11	120.1	C33—C34—C35	122.91 (19)
C11—C12—C13	121.3 (2)	C33—C34—H34	118.5
C11—C12—H12	119.3	C35—C34—H34	118.5
C13—C12—H12	119.3	C30—C35—C34	119.7 (2)
C14—C13—C12	120.0 (2)	C30—C35—H35	120.1
C14—C13—H13	120.0	C34—C35—H35	120.1
C12—C13—H13	120.0	C37—C36—C28	113.77 (14)
C13—C14—C9	119.97 (19)	C37—C36—H361	108.8
C13—C14—H14	120.0	C28—C36—H361	108.8
C9—C14—H14	120.0	C37—C36—H362	108.8
C16—C15—C7	115.08 (13)	C28—C36—H362	108.8
C16—C15—H151	108.5	H361—C36—H362	107.7
C7—C15—H151	108.5	C38—C37—C42	117.8 (3)
C16—C15—H152	108.5	C38—C37—C36	119.2 (2)
C7—C15—H152	108.5	C42—C37—C36	122.99 (19)
H151—C15—H152	107.5	C37—C38—C39	118.7 (3)
C17—C16—C21	116.76 (19)	C37—C38—H38	120.7
C17—C16—C15	120.24 (18)	C39—C38—H38	120.7
C21—C16—C15	123.00 (18)	C40—C39—C38	121.7 (3)
C16—C17—C18	120.4 (2)	C40—C39—H39	119.1
C16—C17—H17	119.8	C38—C39—H39	119.1
C18—C17—H17	119.8	C41—C40—C39	119.5 (3)
C19—C18—C17	122.5 (3)	C41—C40—H40	120.2
C19—C18—H18	118.7	C39—C40—H40	120.2
C17—C18—H18	118.7	C40—C41—C42	119.5 (3)
C20—C19—C18	118.1 (2)	C40—C41—H41	120.3
C20—C19—H19	120.9	C42—C41—H41	120.3
C18—C19—H19	120.9	C41—C42—C37	122.8 (3)
C19—C20—C21	119.8 (3)	C41—C42—H42	118.6
C19—C20—H20	120.1	C37—C42—H42	118.6

### Hydrogen-bond geometry (Å, °)

**Table:**

*D*—H···*A*	*D*—H	H···*A*	*D*···*A*	*D*—H···*A*
C10—H10···O1	0.93	2.19	2.817 (3)	124
C14—H14···O2	0.93	2.34	2.996 (2)	127
C31—H31···O4	0.93	2.47	3.107 (2)	126
C35—H35···O3	0.93	2.37	2.989 (3)	124
C11—H11···O4	0.93	2.48	3.404 (3)	175
O2—H2O···O1^i^	0.90 (2)	1.88 (2)	2.769 (2)	174 (2)
O4—H4O···O3^ii^	0.98 (2)	1.82 (2)	2.793 (2)	178 (2)
C24—H24···O1^ii^	0.93	2.48	3.310 (3)	148
C3—H3···O3^iii^	0.93	2.46	3.327 (3)	154
C34—H34···O2^iv^	0.93	2.49	3.415 (3)	176

Symmetry codes: (i) −*x*+1, −*y*, −*z*+1; (ii) −*x*, −*y*+1, −*z*+1; (iii) −*x*+1, −*y*+1, −*z*+1; (iv) *x*, *y*+1, *z*.

## References

[bb1] Ali, S. A. & Wazeer, M. I. M. (1988). *J. Chem. Soc. Perkin Trans. 1*, pp. 597–605.

[bb2] Altomare, A., Burla, M. C., Camalli, M., Cascarano, G. L., Giacovazzo, C., Guagliardi, A., Moliterni, A. G. G., Polidori, G. & Spagna, R. (1999). *J. Appl. Cryst.* **32**, 115–119.

[bb3] Astolfi, P., Bruni, P., Greci, L., Stipa, P., Righi, L. & Rizzoli, C. (2003). *Eur. J. Org. Chem.* pp. 2626–2634.

[bb4] Balasubramanian, N. (1985). *Org. Prep. Proced. Int.* **17**, 23–47.

[bb5] Belletti, D., Cantoni, A. & Pasquinelli, G. (1993). *AED* Internal Report 1/93. Centro di Studio per la Strutturistica Diffrattometrica del CNR, Parma, Italy.

[bb6] Bernotas, R. C., Sabol, J. S., Sing, L. & Friedrich, D. (1999). *Synlett*, **5**, 653–655.

[bb7] Bernstein, J., Davis, R. E., Shimoni, L. & Chang, N.-L. (1995). *Angew. Chem. Int. Ed. Engl.* **34**, 1555–1573.

[bb8] Bond, C. C. & Hooper, M. (1974). *Synthesis*, p. 443.

[bb9] Bruni, P., Giorgini, E., Tommasi, G. & Greci, L. (1998). *Tetrahedron*, **54**, 5305–5314.

[bb10] Chiacchio, U., Saita, M. G., Crispino, L., Gumina, G., Mangiafico, S., Pistara, V., Romeo, G., Piperno, A. & De Clercq, E. (2006). *Tetrahedron*, **62**, 1171–1181.

[bb11] Farrugia, L. J. (1997). *J. Appl. Cryst.* **30**, 565.

[bb12] Greci, L., Tommasi, G., Bruni, P., Sgarabotto, P. & Righi, L. (2001). *Eur. J. Org. Chem.* pp. 3147–3153.

[bb13] Janzen, E. G. (1971). *Acc. Chem. Res.* **4**, 31–40.

[bb14] Jones, R. C. F., Martin, J. N. & Smith, P. (2000). *Synlett*, **7**, 967–970.

[bb15] Keller, E. (1997). *SCHAKAL97* University of Freiburg, Germany.

[bb16] Merino, P. (2005). *Compt. Rend. Chim.* **8**, 775–788.

[bb17] Nardelli, M. (1995). *J. Appl. Cryst.* **28**, 659.

[bb18] Pisaneschi, F., Della Monica, C., Cordero, F. M. & Brandi, A. (2002). *Tetrahedron Lett.* **43**, 5711–5714.

[bb19] Revuelta, J., Cicchi, S., de Maijere, A. & Brandi, A. (2008). *Eur. J. Org. Chem.* pp. 1085–1091.

[bb20] Sheldrick, G. M. (2008). *Acta Cryst.* A**64**, 112–122. 10.1107/S0108767307043930 18156677

[bb21] Tommasi, G., Bruni, P., Greci, L., Sgarabotto, P. & Righi, L. (1999). *J. Chem. Soc. Perkin Trans. 1*, pp. 681–686.

[bb22] Yamada, F., Kawanishi, A., Tomita, A. & Somei, M. (2003). *Arkivoc*, **viii**, 102–111.

[bb23] Zubarev, V. E. (1979). *Russ. Chem. Rev.* **48**, 1361–1392.

